# Developing a Decision-Aid Website for Breast Cancer Surgery: An Action Research Approach

**DOI:** 10.2196/10404

**Published:** 2019-02-04

**Authors:** Yu-Ting Hung, Ching-Fang Wu, Te-Hsin Liang, Shin-Shang Chou, Guan-Liang Chen, Pei-Ni Wu, Guan-Rong Su, Tsuey-Huah Jang, Chang-Yi Liu, Ching-Yen Wang, Ling-Ming Tseng, Shuh-Jen Sheu

**Affiliations:** 1 School of Nursing National Yang-Ming University Taipei Taiwan; 2 Public Health Center of Taoyuan District Department of Public Health Taoyuan City Government Taoyuan Taiwan; 3 Infinity Clinic Taipei Taiwan; 4 Department of Statistics and Information Science Fu Jen Catholic University New Taipei City Taiwan; 5 Taipei Veterans General Hospital Taipei Taiwan; 6 National Taiwan University Hospital Taipei Taiwan; 7 Cathay General Hospital Taipei Taiwan; 8 AdvancedTEK International Corp Taipei Taiwan; 9 Infusion Treatment Center at Cancer Center South Bay, Stanford Healthcare San Jose, CA United States; 10 Comprehensive Breast Health Center & Division of General Surgery Department of Surgery Taipei Veterans General Hospital Taipei Taiwan; 11 Department of Surgery School of Medicine National Yang-Ming University Taipei Taiwan; 12 Institute of Community Health Care School of Nursing National Yang-Ming University Taipei Taiwan

**Keywords:** breast cancer, surgery-related decision making, website, action research

## Abstract

**Background:**

Patients with early-stage breast cancer have numerous options when choosing the type of breast surgery method to be applied. Each of these options lead to a similar long-term survival rate, but result in significant differences in appearance, function, cost, recurrence rate, and various other relevant considerations. However, the time available for detailed communication with each patient is often limited in clinics, which puts these women under great psychological stress and can hinder their surgery-related decision making.

**Objective:**

The objective of this study was to develop a multipurpose surgery decision-making website providing medical information, psychological support, and decision-related simulation for women during breast cancer surgery-related decision making.

**Methods:**

Using the 4 steps of action research, which involve multigroup teamwork via regular team meetings, the following were performed: (1) Planning: searching, analyzing, and evaluating health websites to consensually decide the major infrastructure; (2) Action: work was performed simultaneously in 4 groups, which consisted of medical information collection and editing, patient interviews and data extraction, webpage content design, and programming to create or host the website; (3) Evaluation: the website was tested by clinical experts and focus groups of former breast cancer patients to assess its effectiveness and pinpoint appropriate improvements; and (4) Reflection: constant dialogue was conducted between the various participants at each step, which was used as the foundation and motivation of next plan-action-evaluation-reflection circle.

**Results:**

Using the action research approach, we completed the development of our website, which includes the following: (1) “Woman’s Voice”—an animated comic depicting the story of a female breast cancer patient with interspersed questions for the users that will help them better empathize with the experience; (2) “Cancer Information Treasure House”—providing breast cancer surgery-related information through text, tables, pictures and a presentation video; (3) “Decision-making Simulator”—helping patients think through and check the pros and cons of the different surgical options via visual-based interactions including “Stairs Climbing” and “Fruit of Hope”; and (4) “Recommended Links”—providing reliable websites for further reference. Additionally, we have further improved the website based on the feedback received from postsurgery breast cancer patients and clinicians. We hope to continue improving to better meet both the patients’ and health providers’ needs and become a practical decision-making aid for patients undergoing breast cancer surgery.

**Conclusions:**

We have created the first breast cancer surgery decision-making assistance tool in Taiwan using a “Web-based” and multifunctional website design. This site aims to provide health care knowledge, psychological healing, and emotional support functions, as well as decision-making capability enhancement simulations. We look forward to assisting breast cancer patients in their decision-making process and expect our website to increase patient’s autonomy and improve their communication with clinicians.

## Introduction

In Asia, as in other parts of the world, Cancer is one of the major health concerns. Different types of cancer have different incidence rate according to gender, age, and other demographic factors. Among the different types, breast cancer has the highest incidence rate among the female population of Taiwan. According to the 2017 report of the Taiwan Health Promotion Administration Ministry of Health and Welfare, the incidence rate of breast cancer was 125.64 per 100,000. This means that in 2015, the year for which the latest statistics are available, 14,801 women were diagnosed with breast cancer. Among these women, roughly 80% had been diagnosed with early-stage breast cancer [1]. The key point is the fact that patients with early-stage breast cancer have more than one treatment choice, but unfortunately, they do not necessarily have all the information needed to make an informed decision regarding the kind of treatment best suited to their particular case. For instance, lumpectomy and mastectomy are often recommended for most patients with early-stage breast cancer [2]. In addition, breast reconstruction following mastectomy is another choice. It is worth noting that there is a general consensus among the medical community that the long-term survival rate for this disease is very similar across these 3 different types of surgery [3-6]. Thus, if the tumor size and location allow, patients are able to choose their preferred surgical intervention.

Patients’ decisions about breast cancer surgery are multifactorial, and their understanding of the disease and its treatment affects their decision making [7]. Our initial empirical observations, which were later confirmed, suggested that 4 important factors hinder Taiwanese breast cancer patients’ ability to make well-informed decisions regarding the kind of treatment best suited to their particular case. First, there is a lack of easily available information. Studies have shown that when facing decision making regarding breast cancer surgery, patients often require more information to decide which type of breast surgery is suitable for them [8,9]. However, this requirement for information is mostly unmet; they do not fully understand the different surgical procedures that might apply to their case [10,11]. Second, there are negative emotions resulting from their personal experience of facing the diagnosis. Pretreatment symptoms in newly diagnosed patients include not only cognitive but also emotional aspects; patients often experience a variety of negative emotions due to a link between cancer and death [12-14], in particular, high levels of anxiety and uncertainty [14,15]. Third, another key factor hindering the patients’ ability to make a well-informed decision regarding the kind of surgery most suitable for them is the limited clinic time available or, in other words, the relatively short time span of their appointments with their physician. Finally, there is a significant cultural factor that also affects the decision-making process of patients with breast cancer in Taiwan, namely local taboos regarding certain medical conditions. Talking about breast cancer, as well as other conditions related to the intimate parts of one’s body, is not encouraged in Chinese culture. Therefore, women tend to be hesitant even when talking to their physicians, which affects their requesting of further information regarding their condition, disclosing their opinion, and showing their personal feelings when required.

Decision aids are tools intended to help patients make an informed decision, and they do this by providing them with better knowledge and a general overview of all the options available to them with the pros and cons of each one. By adding clarity and congruence between decision and personal values, decision aids help reduce conflict during the decision-making process, as well as helping patients break away from passiveness [16]. A key aspect of this research is the creation of a website that relies on a set of audiovisual and digital processes to aid patients make an appropriate decision regarding breast cancer. Compared with the traditional alternatives, the advantages of digital and audiovisual tools are that they provide better and more vivid visual effects. By emphasizing interactive features and varied visual stimuli, patients are able to obtain first-hand and immediate information. The digital nature of this site will facilitate the ability for the information to be updated frequently, which will provide the patients with the most reliable knowledge available at a given moment [17]. In a face-to-face consultation between a physician and a patient, the use of computer-based decision aids can increase the efficiency of counseling [18].

Therefore, this study aims to develop a multipurpose surgery decision-making website that acts as a decision-aid tool by providing medical information, psychological support, and decision simulation for patients with breast cancer who are undertaking the decision-making process regarding future surgery.

## Methods

### Research Design

In this research, we applied an action research approach, which is defined as “a disciplined process of inquiry conducted by and for those taking the action. The primary reason for engaging in action research is to assist the ‘actor’ in improving and/or refining his or her actions.” [19] Compared with conventional research methods, one of the key differences is the fact that the main goal of action research is to generate knowledge and solve problems through direct involvement of the actors [20]. In recent years, action research has used tackled topics such as (1) Cross-border Peer Health Educator Programs and Latina/Family Experiences; (2) Psychosocial Needs Post-Genetic Cancer Risk Assessment; and (3) finding positive effects in breast cancer patient/family education [21-23]. However, a limited number of breast cancer-related studies are involved in the decision-making and research processes. Therefore, the ultimate goal of this study is to argue in favor of the benefits to patients and families of a Web-based platform that assists patients with breast cancer in the decision-making process; we concluded that an action research approach is the most suitable way of conducting this investigation.

**Figure 1:**
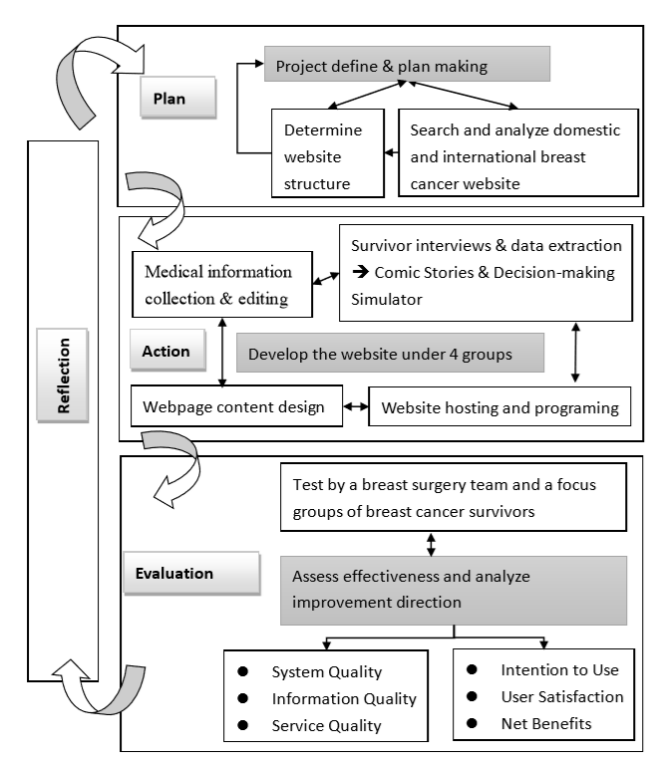
Conceptual framework of the action research.

To achieve our goal, the research was designed to be performed by multiple groups, which were brought together to form an effective work team. Meetings were conducted on a regular basis, namely every 2 weeks during a semester and weekly during the summer and winter breaks (Table 1 provides a detailed account of the 90 meetings that were conducted). The aim was to develop a well-coordinated teamwork system that allowed the whole team to be able to review and adjust their assignments appropriately. During these regular meetings, each group reported on their progress, shared their experiences, and provided mutual feedback. Through the accumulated knowledge and experiences of each group in their designated area, we were able to complete the construction of the proposed website, which is shown in the conceptual framework presented in Figure 1.

### Step 1: Plan

The research team included the participation and expertise of a leading academic scholar (Prof Shuh-Jen Sheu), as well as involvement of significant contributions from several nursing doctoral and master degree candidates, an information technology (IT) engineer, and 3 computer graphics designers; in total, there were 13 team members. During this stage of the project, team members were tasked with the collection and analysis of information related to existing dedicated breast cancer websites. Based on the gathered information and the research conclusions drawn from this information, together with the assistance of the IT specialist, the team undertook the task of creating a website that is central to this investigation.

**Table 1:** Important meeting dates.

Year	Meeting dates	Times (n)
2010	09/21, 10/7, 10/21, 11/16, 12/7, 12/28	6
2011	01/20, 03/31, 04/19, 05/09, 05/30, 06/20, 06/30, 07/07, 07/15, 07/21, 07/28, 08/11, 08/18, 08/24, 09/15, 09/29, 10/25, 10/27, 10/31, 11/10, 11/24, 11/25	22
2012	01/16, 02/23, 03/08, 03/22, 04/05, 04/19, 05/03, 05/17, 05/31, 06/14, 06/28, 11/29, 12/13	13
2013	01/17, 01/29, 02/05, 02/25, 03/11, 04/01, 04/22, 05/23, 05/30, 06/06, 09/27, 10/11, 11/08, 11/22, 12/06, 12/27,	16
2014	01/10, 02/19, 03/17, 03/31, 05/26, 06/09, 06/25, 07/09, 07/16, 08/06, 08/20, 09/01, 09/09, 09/19, 09/26, 10/09, 10/16, 11/13, 10/30, 11/27, 12/11, 12/16, 12/18, 12/25	24
2015	01/20, 01/27, 02/03, 02/10, 03//03, 3/6, 3/10, 3/13, 3/24	9

### Step 2: Action

At this stage of the study, we formed 4 group work teams, each of which tackled different parts of the investigation. Through regular exchange between the 4 groups, various processes within the target areas were presented, analyzed, and corrected by the groups when necessary.

The mission of the 4 groups was as follows:

- *Group 1: The Collecting and Editing of Medical Information*. This group was responsible for gathering and reviewing of existing breast cancer surgery literature with the aim of creating easy-to-understand formats and simple graphics renderings.
- *Group 2: Patients Interviews and Data Extraction*. This group was responsible for interviewing women who had undergone breast cancer surgery in order to collect information related to their decision-making experiences. The gathered information was then analyzed through qualitative content analysis to understand patients’ mindsets vis-à-vis the decision-making process; these were then used to identify any possible patterns. The mindset and possible patterns would become an important feature of the Animated Comics and the Animated Decision-making Simulator on our website.
- *Group 3: Webpage Content Design*. This group was responsible for webpage design, which included the Animated Comics, Simulation Games, Medical Information Display, and Relevant Links Page. This content was created using various graphics software including Adobe Illustrator CS6 (Adobe Incorporated) and Photo Impact X3 (Ulead Systems Incorporated).
- *Group 4: Programming and Website Hosting*. This group was responsible for informatics work related to program coding and the hosting of the website. The tech team used ActionScript technology for front-end animation programs and J2EE technology for back-end animation programming. The operating system was Ubuntu 10 (Canonical Ltd), while the network server was Apache Tomcat 7 (Apache Software Foundation). The Web-based machine was hosted by the Amazon’s EC2 Cloud Service (Amazon.com Inc). The internet domain name was accessed through Seed net (Software Engineering Environment Development Network). In addition, various program development tools were used including Eclipse Java EE IDE (Eclipse Foundation) for Web Developers, Oracle Java SE Development Kit 7 (Oracle Corporation), and Flash Develop 4 (Flash Develop Team) .

### Step 3: Evaluation

On completion of the preliminary website, we used a multifaceted approach to evaluate the website. The participants in the evaluation consisted of 9 former breast cancer patients; 12 clinical staff members comprising surgeons, case managers, nurse specialists, and oncology radiologists; and 19 research team members, including academic scholars, doctoral students, master students, undergraduate students, information engineers, and graphic designers. The website evaluation was conducted using three different processes: individual interviews and focus groups with former breast cancer patients, individual and group consultations with the clinical staff, and an internal evaluation via regular team meetings. Any relevant information was recorded and logged via minutes of meeting.

### Step 4: Reflection

Reflection consisted of the constant dialogue among team members during every stage of the investigation process. Team members communicated with each other and shared their views from their diverse perspectives. This allowed the researchers to review their role in retrospective during this action research and to develop different patterns of thinking related to the health care needs of patients with breast cancer during the surgery-related decision-making process. This, in turn, became the foundation and motivation for the next plan-action- evaluation-reflection circle.

## Results

### Patients Interviews and Data Extraction

This survey took place at two different medical centers in Taipei City from September 2010 through January 2012. A total of 31 women who were being treated for breast cancer were interviewed, including 11 who underwent mastectomy, 15 who underwent lumpectomy, and 5 who underwent mastectomy followed by immediate breast reconstruction. Of these patients, 20 were interviewed 1 day before their surgery, 2 while they were under chemotherapy, and 9 after they finished chemotherapy, which had started 6-12 months earlier. Based on the results of the Qualitative Content Analysis, we concluded that “the impact factors for surgery-related decision making,” “the decision-making modes related to the 3 types of breast surgery,” and “the mindset after deciding to undergo breast cancer surgery” were the most suitable areas that could function as the basis of our webpage content and help with developing the Animated Comics and Decision-making Simulator.

### Webpage Content Design

After a broad collection of information and its analysis by the various work groups, we concluded that the information available on breast cancer surgery-related decision making and on the psychological support of patients with breast cancer on Taiwanese websites was insufficient for local patients with breast cancer. Based on this premise, we adopted a multifaceted approach to design and construct a website that was more interactive and individualistic; the aim being to fulfill this information need. Our final proposal was a breast cancer surgery decision-making support website based on a decision-making assistant, together with appropriate psychological support. The contents of the website are as follows.

#### Website Structure

**Figure 2:**
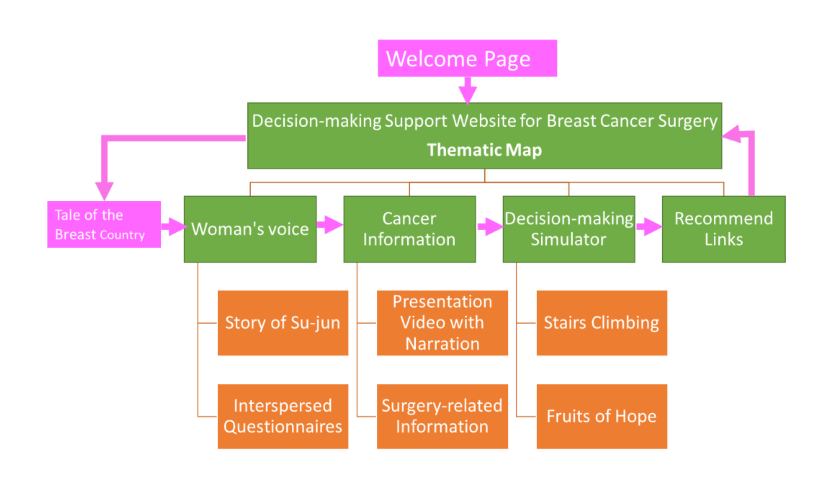
Website structure.

Upon entering the welcome page, patients are shown a thematic map entitled “Tale of the Breast Country.” Underneath this map, our webpage follows a logical structure where the 4 main thematic parts with their corresponding submenus are displayed as the user clicks on them. The 4 main themes are Women’s Voice, Cancer Information Treasure House, the Decision-making Simulator, and Recommend Links (Figure 2).

#### Thematic Map

**Figure 3:**
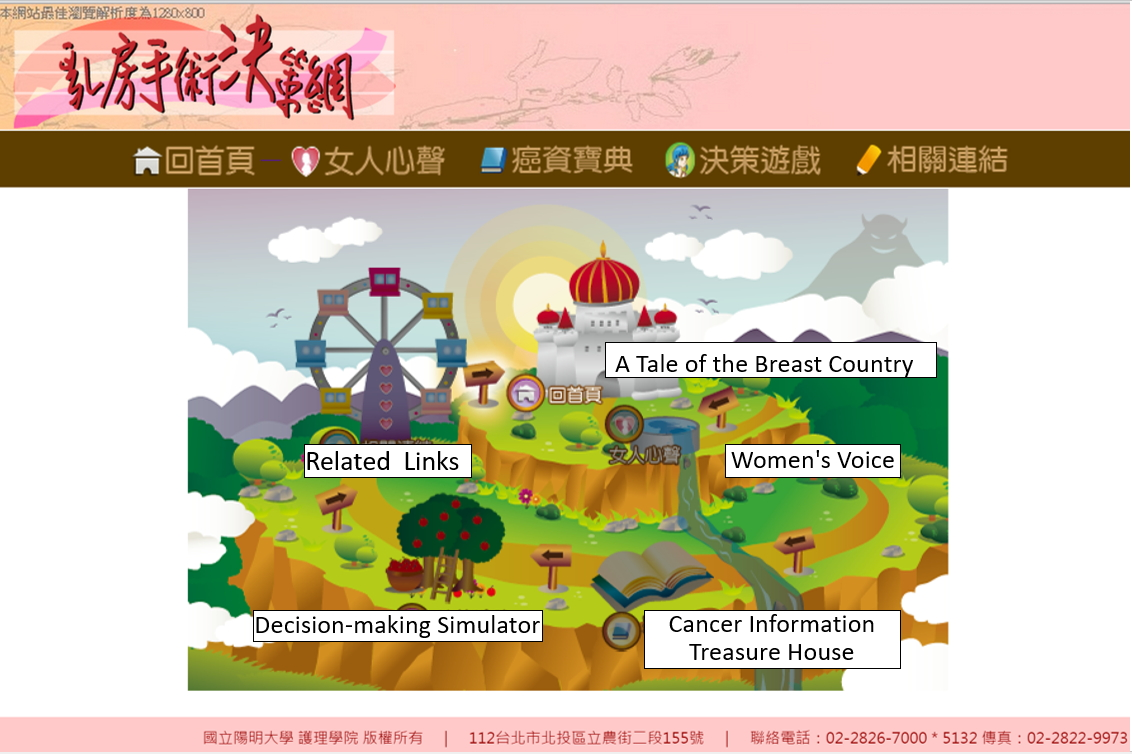
Thematic map.

In addition to displaying a simple text menu on the top of the page, we also created a thematic map (Figure 3) using colorful flash animations to help users understand the main themes and the whole structure of the website.

#### A Tale of Breast Country

**Figure 4:**
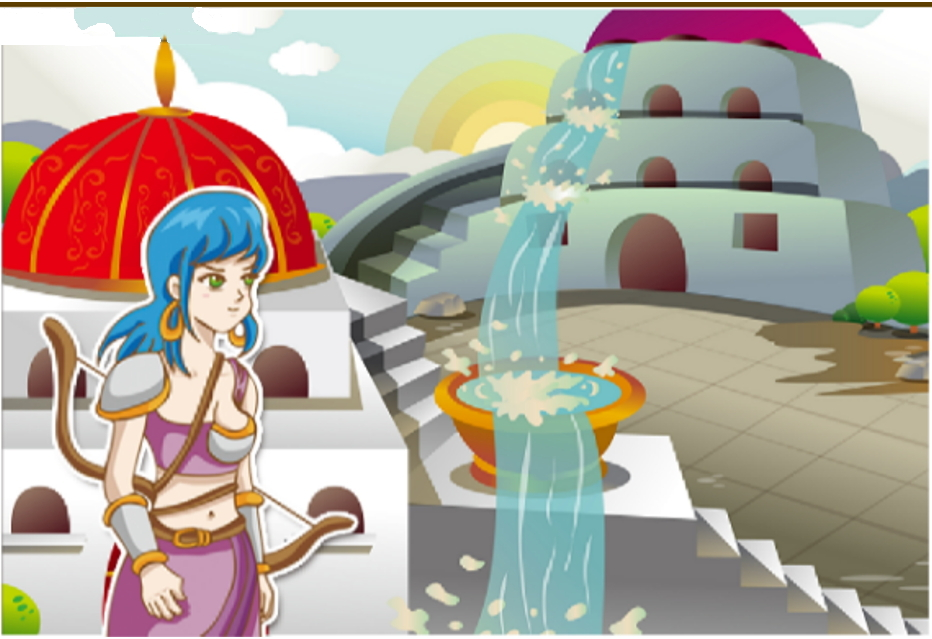
Tale of Breast Country.

We created an animated story “Tale of the Breast Country,” which describes how women in Breast Country were attacked by the evil of Adenocarcinoma, which led to the development of breast cancer. The main character is the female warrior Maya (primarily adopted from legendary Greek Amazon who cut away her left breast to use her bow and arrows more effectively) who stands up for the inhabitants of the country and seeks ways to fight this problem. Her adventures are a descriptive process of the various possible approaches to dealing with breast cancer. The different paths that her journey takes form the basis of part of the website (Figure 4).

#### Women’s Voice

**Figure 5:**
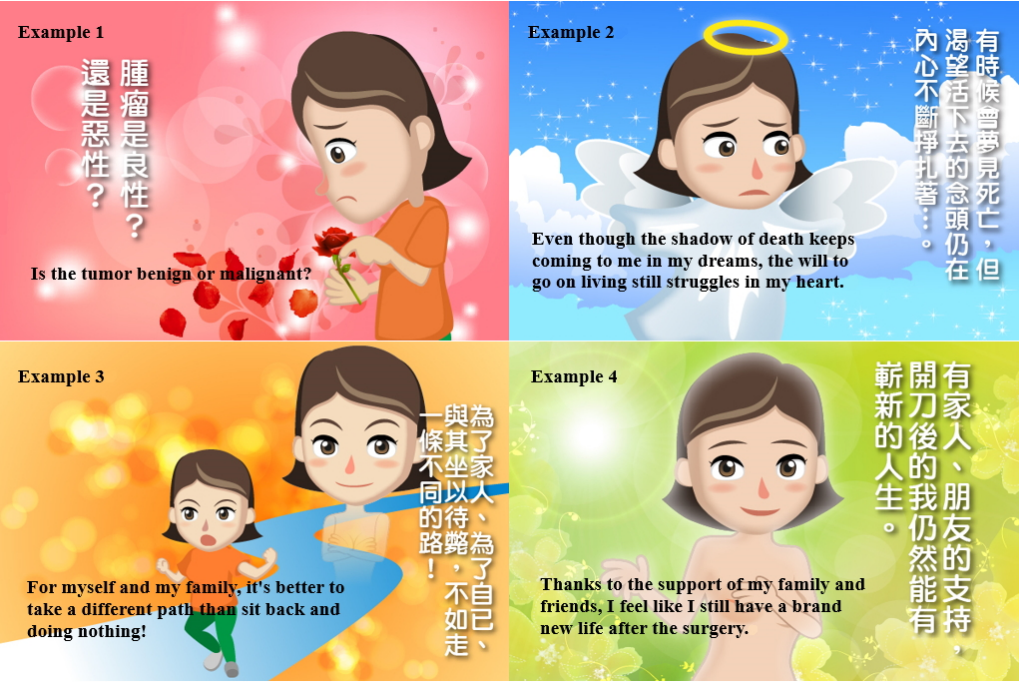
Four examples of women's voice.

We created an animated story based on the results obtained from analyzing our patient interviews, and these focused on “the mindset after deciding to opt for breast cancer surgery.” This story was named the “Story of Shu-Jun” and tells the story of Shu-Jun (a common Chinese female first name), who is a woman full of despair and anxiety after realizing she had developed cancer. She represents the various emotional reactions that women tend to show when diagnosed with this condition, such as fear of death and concerns about treatment options. At the end of the story, our heroine finds the courage to face the disease. Overall, 32 animated videos are displayed covering 8 different topics, which means that each topic consists of 4 animated comics. These 4 animated comics are constructed on the basis of the parameters of “the mindset after deciding breast cancer surgery.” Each comic is accompanied by a female narration and soft background music; they are interspersed with questions designed to help patients improve their reaction to matters that create uncertainty and that need psychological adjustment; the aim is to lead patients toward a more natural expression of their personal and psychological feelings (Figure 5).

#### Cancer Information Treasure House

**Figure 6:**
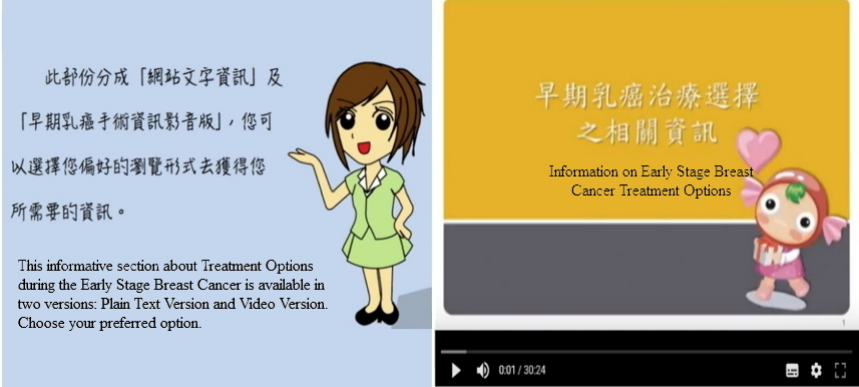
Cancer information treasure house.

We gathered and analyzed a wide array of information that might be helpful to patients during the breast cancer surgery decision-making process, and this information was edited into 7 topics that are presented in a Chinese homophonic or symbolic way. These include the following: basic knowledge of breasts and breast cancer, risk factors, diagnostic examinations to identify breast cancer, different stages of breast cancer, various treatments available for breast cancer, various factors related to the prognosis of breast cancer, and care after breast cancer surgery. In addition, we provide presentation videos as different choices available using text and pictures covering the 7 topics (Figure 6).

#### Decision-Making Simulator

“Stairs Climbing” and “Fruits of Hope” are the 2 breast surgery simulation tools available on our platform; they are intended to help patients to think through the process from different perspectives. These tools were crafted on the basis of the qualitative analysis of the interviews conducted with the postsurgery breast cancer patients. The qualitative analysis was conducted focusing on two key aspects, namely “the impact factors of surgery-related decision making” and “decision-making modes regarding the 3 types of breast surgery.”

**Figure 7:**
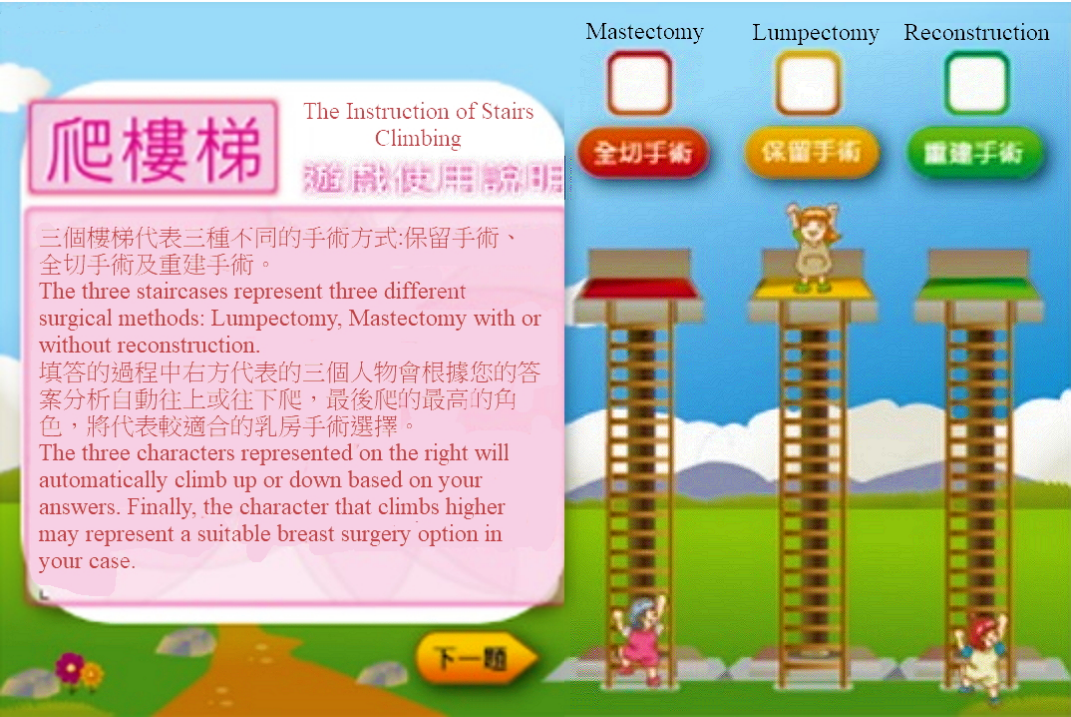
Stairs Climbing.

In “Stairs Climbing” (Figure 7), the left part shows the instructions and 24 questions related to “the factors that have an impact on the surgery-related decision-making process.” On the right side, there are 3 stairs with 3 characters, which represent the 3 types of breast cancer surgery. By choosing Strongly Agree, Agree, No Opinion, Disagree, and Strongly Disagree, the 3 characters change their hierarchical position on the stairs according to the user’s answer choices. Following this logic, the character that climbs to the top at the end of the questionnaire represents the surgery that seems to be preferred by the patient.

**Figure 8:**
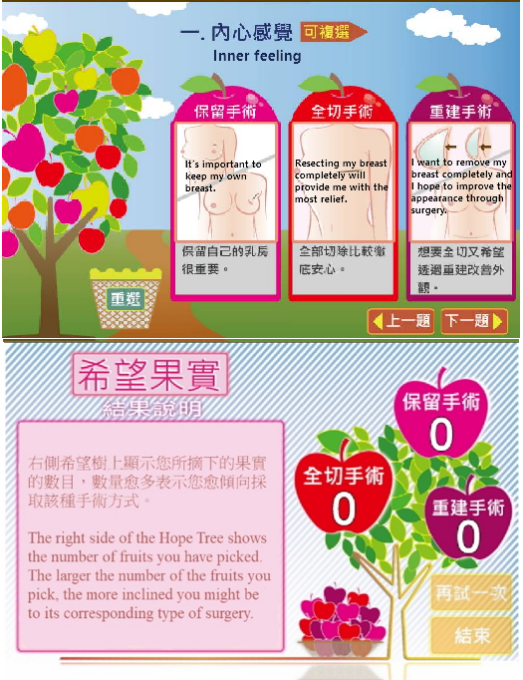
Fruits of Hope.

“Fruits of Hope” (Figure 8) is the second tool designed on the basis of the qualitative analysis of the interviews with postsurgery breast cancer patients. This tool was specifically based on the interview’s responses regarding the “decision-making modes of the 3 types of breast surgery.” Nine aspects of the surgical outcomes are applied as follows: personal feeling, recurrence rate, postoperative appearance, body balance, clothing choices, sex and intimacy, therapeutic category, clinic numbers, and economic considerations. There are fruits of different sizes and colors on a “Hope Tree” that reflect the consequences of the different surgeries. Users can choose different surgical outcomes by clicking on the pictures to pick the fruits. The fruits are then counted by color, and the result is shown at the end of the simulation. Users are told if the choice can meet their needs at the outcome level.

To avoid conflicts or confusion after using the tools, detailed instructions are provided, at the beginning of the process, during the process, and at the end of the simulation page. It is worth remembering that the main purpose of these 2 simulations is to assist patients in need of further information and to allow them to make well-informed decisions, rather than replacing entirely the decision-making process or making the decision for them. Furthermore, it is clearly stated that the results of the simulation only reflect an individual’s values and preferences. Patients are reminded that it is still necessary to consult with their surgeon before making a final decision.

### Related Links

As the main purpose of this work is the creation of a decision-making aid website for patients with breast cancer, we have also collected a list of related local and foreign websites that will be able to help patients as reference points and allow them to make comparisons between options.

Local websites are as follows:

- Taiwan Breast Cancer Foundation
- Taiwan Breast Cancer Alliance
- Taichung Kaihuai Association
- Taipei warm Association
- Taiwan Breast Reconstruction Society
- Kaohsiung Heart to Hand Association
- Global Chinese Breast Cancer Organizations Alliance
- HER2 Club
- Breast Center of Taiwan Adventist Hospital

Foreign websites are as follows:

- Breast Cancer Care
- Breastcancer.org
- Bresdex

**Table 2:** A list of clinical staff members who participated in the study.

Office unit	Date of participation	Form of participation	Job title
Medical center	04/17/2015	Breast cancer multispecialty medical team joint discussion	3 Surgeons; 1 General surgery chief resident; 1 Radiation oncology physician; 2 Breast cancer case manager; 1 Radiologist; 1 Pathology physician
Medical center	08/2010-12/2014	Participated in research team meetings	1 Surgical nurse practitioner
Medical center	01/27/2015	Participated in the focus group	1 Surgical nurse practitioner
Medical center	10/19/2014	Participated in research team meeting	1 Breast cancer case manager
Regional hospital	07/15/2015	Individual consultation	1 Assistant of head of the surgical department

### Website Evaluation

To further evaluate the functioning and effectiveness of our website, 3 main approaches were used during the evaluation process. First, we performed internal assessments through regular team meetings. Second, we conducted several interviews with 3 individual patients and with focus groups consisting of 7 patients. We tested our website’s logics and functions with these former breast cancer patients about their experiences regarding the process of deciding the type of breast cancer surgery best suited for them. The clarification and verification took place in one medical center in Taipei City, from December 2014 through March 2015. Third, we formed a group consisting of 12 clinical staff members, namely surgeons, case managers, nurse specialists, and oncology radiologists. We then demonstrated our website and conducted individual and group consultations with this medical team. Table 2 lists the clinical staff partaking in this group.

## Discussion

### Principal Findings

Past studies in western literature have highlighted the importance of the principles of informed consent and patient autonomy; however, the medical literacy of patients, their preferences, as well as a close relationship (mutual communication and understanding) between patients with breast cancer and their surgeon, are often key challenges for women while making a meaningful decision regarding their impending breast surgical choice. Data analysis of the first encounter of patients with their surgeon has indicated the high level of reassurance patients obtain from the authority and expertise they see in the surgeon figure [24]. From a clinician’s point of view, this situation is of decisive importance to patients with breast cancer; however, we found that what the patients want and what the clinicians deem necessary in terms of the website content can often be conflicting. For instance, some clinicians believe that what a patient needs during decision making is medical knowledge as opposed to reading comics, while almost all participants in breast cancer support groups felt very touched after watching the Story of Shu-Jun because they can relate the animated comic to their own experiences. In addition, the question helped them express their current feelings, and the last comic topic “revival” made them feel calmer and full of hope when facing upcoming treatment. This work applies action research, which aims to bridge the gap between patients and clinicians and caters more toward the feelings and needs of a patient’s mind and body than previous approaches.

Studies have often demonstrated the positive effects of Web-based decision-making tools [25]. Considering this, our research is aimed to more closely identify with and assure patients during the dynamic relationship that is patient-doctor communications. During the process of developing the website, the requirement to balance patients’ needs and clinicians’ professional concerns became a problem that needed a solution. After conducting a thorough literature review and discussing the problem during the research meetings of the work groups, we finally came to an agreement regarding the actions needed. First, the team would closely collaborate with professionals in medical centers; this would allow the updating and releasing of medical information onto the website quickly to ensure consistency of the information. Second, the medical team would evaluate the website before referring it to patients. In parallel, we emphasized the fact that the site had an assisting role and it was not intended to replace the professional clinician’s assessment. Furthermore, we would continue to evaluate the effectiveness of the Web-based tool for women who are newly diagnosed breast cancer using experiences by designing more comprehensive quantitative and qualitative research to put in place subsequent relevant modifications and various promotional plans. This would continue the cycle of action research into the future based on any new results.

We consider that the decisions of patients regarding breast cancer surgery are affected by multiple factors, including the reliability of the information available, availability of information, patients’ emotional needs, the fact that clinical counseling time is limited, and the effect of patients’ culture on the process. Therefore, to meet the needs of early-stage breast cancer-related decision-making process, this study was designed to use a multifaceted approach.

Web-based breast cancer decision aids have become increasingly common in recent years [26-30]. The research design we used was an action research approach. The core ideas of applying action research involve practical reflection and real performance during the investigation process, critique liberation, building investigation strategies, and research innovation [31]. Compared with the development of medical information websites, in general, our Web-based breast cancer decision aid development process has been more systematic and sophisticated and has improved on previous sites primarily by learning about the patients’ perspectives and needs. To get closer to the experience of women, we primarily used qualitative interviews and content analysis. In addition, we used a multifaceted approach to verify the meaning and functioning of the website. These distinct approaches had the same objectives, which was to meet the needs and special conditions associated with local Taiwanese cases. This study serves as a reference for describing how to develop decision support tools for women with breast cancer by considering their own corresponding culture and the region in which they live.

### Limitations

We faced many challenges during website construction for this study; they included language, communication, information engineering, and art design, as well as how to train the decision-making ability. In addition to the type of tumor and its stage being different for each patient, opinions and expert advice may also differ between clinicians and hospitals. Detailed individual treatment information, such as recurrence rate, survival rate, treatment plan, and reconstructive decisions, is still dependent on the physician’s assessment, which is also dependent on their experience and personal opinions, rather than what is described on the site. Hence, we can only recommend patients to find more information and discuss their personal issues with their surgeon directly. Finally, the technique of website hosting, programming, and patterns design depends on external technical support. The action research cycles provide an opportunity to modify and confirm this repeatedly, as it is quite a repetitive and lengthy process for the professionals involved. This also means that maintaining long-term collaborations with various professionals to maintain these cycles requires a considerable budget. Therefore, it has been suggested that more students and teachers in IT engineering and design-related departments should be trained and invited to work together in this area.

This paper is focused on the construction part, and further detailed quantitative and qualitative studies on the website effectiveness will be the next step. As our search and analysis of the website are limited to a Chinese version, we are, therefore, unable to explore the advantages and disadvantages related to non-Chinese and non-English literature and websites. However, this study can serve as a significant foundation and reference point for anyone who is interested in the future in the planning, promotion, operation, and long-term management of similar websites.

### Conclusions

This study aims to build up a dedicated decision-aid website with a good structure and useful content so that it can help women who are facing the dilemma of choosing the right breast cancer surgery for them. As the first surgery decision-making assistance website in Taiwan, the content of this site provides not only health care knowledge but also psychological healing functionality and decision-making simulation. Using the action research approach, we gathered breast cancer survivors, clinicians, nursing scholars, nursing students, information engineers, and computer graphics designers together. With their combined knowledge and expertise, this team managed effectively to shorten the distance between theory and practice. After a rigorous process of investigation, analysis, and testing, we have successfully completed the development of our surgery decision-making website for patients with breast cancer. Through testing of our website via the focus group and further interviews, we have concluded that this kind of work can indeed help patients with early-stage breast cancer make a more informed decision regarding the type of surgery they would prefer to undergo. We look forward to assisting patients with breast cancer in their decision-making process. We expect that this will increase the autonomy of these patients and improve communication with their clinicians.

## Abbreviations

IT: information technology

## References

[1] (2017). Health Promotion Administration, Ministry of Health and Welfare.

[2] Gradishar WJ, Anderson BO, Balassanian R, Blair SL, Burstein HJ, Cyr A, Elias AD, Farrar WB, Forero AD, Giordano SH, Goetz MP, Goldstein LJ, Isakoff SJ, Lyons J, JE PK, Mayer IA, McCormick B, Moran MS, O'Regan RM, Patel SA, Pierce LJ, Reed EC, Salerno KE, Schwartzberg LS, Sitapati AD, Smith KL, Smith ML, Soliman HY, Somlo G, Telli M, Ward JH, Shead DA, Kumar R (2017). NCCN Guidelines Insights: Breast Cancer, Version 1.2017. J Natl Compr Canc Netw.

[3] Black DM, Hunt KK, Mittendorf EA (2013). Long term outcomes reporting the safety of breast conserving therapy compared to mastectomy: 20-year results of EORTC 10801. Gland Surg.

[4] Chen H, Wu K, Wang M, Wang F, Zhang M, Zhang P (2017). A standard mastectomy should not be the only recommended breast surgical treatment for non-metastatic inflammatory breast cancer: A large population-based study in the Surveillance, Epidemiology, and End Results database 18. Breast.

[5] Fisher B, Anderson S, Bryant J, Margolese RG, Deutsch M, Fisher ER, Jeong J, Wolmark N (2002). Twenty-year follow-up of a randomized trial comparing total mastectomy, lumpectomy, and lumpectomy plus irradiation for the treatment of invasive breast cancer. N Engl J Med.

[6] Veronesi U, Cascinelli N, Mariani L, Greco M, Saccozzi R, Luini A, Aguilar M, Marubini E (2002). Twenty-year follow-up of a randomized study comparing breast-conserving surgery with radical mastectomy for early breast cancer. N Engl J Med.

[7] Mac Bride MB, Neal L, Dilaveri CA, Sandhu NP, Hieken TJ, Ghosh K, Wahner-Roedler DL (2013). Factors associated with surgical decision making in women with early-stage breast cancer: a literature review. J Womens Health (Larchmt).

[8] Kimiafar K, Sarbaz M, Shahid Sales S, Esmaeili M, Javame Ghazvini Z (2016). Breast cancer patients' information needs and information-seeking behavior in a developing country. Breast.

[9] Littlechild S, Barr L (2013). Using the Internet for information about breast cancer: a questionnaire-based study. Patient Educ Couns.

[10] Liao M, Chen S, Chen S, Lin Y, Hsu Y, Hung H, Wang C, Chen M, Jane S (2012). Changes and predictors of unmet supportive care needs in Taiwanese women with newly diagnosed breast cancer. Oncol Nurs Forum.

[11] Puts MTE, Papoutsis A, Springall E, Tourangeau AE (2012). A systematic review of unmet needs of newly diagnosed older cancer patients undergoing active cancer treatment. Support Care Cancer.

[12] Mastrovito RC (1972). Emotional considerations in cancer and stroke. In cancer. N Y State J Med.

[13] Chappy SL (2004). Women's Experience With Breast Biopsy. AORN Journal.

[14] Liao M, Chen M, Chen S, Chen P (2008). Uncertainty and anxiety during the diagnostic period for women with suspected breast cancer. Cancer Nurs.

[15] Liu L, Li C, Tang ST, Huang C, Chiou A (2006). Role of continuing supportive cares in increasing social support and reducing perceived uncertainty among women with newly diagnosed breast cancer in Taiwan. Cancer Nurs.

[16] Stacey D, Légaré F, Lewis K, Barry MJ, Bennett CL, Eden KB, Holmes-Rovner M, Llewellyn-Thomas H, Lyddiatt A, Thomson R, Trevena L (2017). Decision aids for people facing health treatment or screening decisions. Cochrane Database Syst Rev.

[17] Sheehan J, Sherman KA (2012). Computerised decision aids: a systematic review of their effectiveness in facilitating high-quality decision-making in various health-related contexts. Patient Educ Couns.

[18] Ventura F, Öhlén J, Koinberg I (2013). An integrative review of supportive e-health programs in cancer care. European Journal of Oncology Nursing.

[19] Sagor R (2000). Guiding School Improvement with Action Research.

[20] Stringer E (2013). Action Research.

[21] Crawford J, Frisina A, Hack T, Parascandalo F (2015). A Peer Health Educator Program for Breast Cancer Screening Promotion: Arabic, Chinese, South Asian, and Vietnamese Immigrant Women's Perspectives. Nurs Res Pract.

[22] Espenschied CR, MacDonald DJ, Culver JO, Sand S, Hurley K, Banks KC, Weitzel JN, Blazer KR (2012). Closing the loop: action research in a multimodal hereditary cancer patient conference is an effective tool to assess and address patient needs. J Cancer Educ.

[23] Macdonald DJ, Deri J, Ricker C, Perez MA, Ogaz R, Feldman N, Viveros LA, Paz B, Weitzel JN, Blazer KR (2012). Closing the loop: an interactive action-research conference format for delivering updated medical information while eliciting Latina patient/family experiences and psychosocial needs post-genetic cancer risk assessment. Fam Cancer.

[24] Beesley H, Goodfellow S, Holcombe C, Salmon P (2016). The intensity of breast cancer patients' relationships with their surgeons after the first meeting: Evidence that relationships are not 'built' but arise from attachment processes. Eur J Surg Oncol.

[25] Jimbo M, Kelly-Blake K, Sen A, Hawley ST, Ruffin MT (2013). Decision Aid to Technologically Enhance Shared decision making (DATES): study protocol for a randomized controlled trial. Trials.

[26] Sherman KA, Kilby CJ, Shaw L, Winch C, Kirk J, Tucker K, Elder E (2017). Facilitating decision-making in women undergoing genetic testing for hereditary breast cancer: BRECONDA randomized controlled trial results. Breast.

[27] Roberto A, Colombo C, Candiani G, Giordano L, Mantellini P, Paci E, Satolli R, Valenza M, Mosconi P (2017). Personalised informed choice on evidence and controversy on mammography screening: study protocol for a randomized controlled trial. BMC Cancer.

[28] Coe AM, Ueng W, Vargas JM, David R, Vanegas A, Infante K, Trivedi M, Yi H, Dimond J, Crew KD, Kukafka R (2016). Usability Testing of a Web-Based Decision Aid for Breast Cancer Risk Assessment Among Multi-Ethnic Women. AMIA Annu Symp Proc.

[29] Manne SL, Topham N, D'Agostino TA, Myers VS, Kirstein L, Brill K, Manning C, Grana G, Schwartz MD, Ohman-Strickland P (2016). Acceptability and pilot efficacy trial of a web-based breast reconstruction decision support aid for women considering mastectomy. Psychooncology.

[30] Elkin EB, Pocus VH, Mushlin AI, Cigler T, Atoria CL, Polaneczky MM (2017). Facilitating informed decisions about breast cancer screening: development and evaluation of a web-based decision aid for women in their 40s. BMC Med Inform Decis Mak.

[31] Greenwood D, Levin M (2007). Introduction to Action Research: Social Research for Social Change, Second Edition.

